# Expression of cyclooxygenase-2 (COX-2) in colorectal adenoma in an indigenous African population in northern Nigeria

**DOI:** 10.4102/ajlm.v14i1.2613

**Published:** 2025-07-30

**Authors:** Abdulrazaq A. Jimoh, Zainab A. Adamu, Mumini W. Rasheed, Samuel K. Richard

**Affiliations:** 1 Department of Anatomic Pathology and Forensic Medicine, Faculty of Basic Clinical Sciences, Federal University of Health Sciences, Azare, Nigeria; 2 Department of Pathology, Faculty of Basic Clinical Sciences, Ahmadu Bello University, Kaduna, Nigeria; 3 Department of Anatomic Pathology, Faculty of Basic Clinical Sciences, Federal University Dutse, Jigawa, Nigeria; 4 Department of Pathology, Faculty of Basic Clinical Sciences, University of Abuja, Gwagwalada, Abuja, Nigeria

**Keywords:** Cyclooxygenase-2, expression, colorectal adenoma, Nigeria, Africa

## Abstract

**Background:**

The clinical significance of adenoma is as a result of being a precancerous lesion with long latency, harbouring of invasive carcinoma, bearing similar clinical features with colorectal cancer, and as part of hereditary colorectal cancer syndromes. Over-expression of the cyclooxygenase-2 (COX-2) enzyme has been noticed in adenomas with unfavourable features. However, this information is limited in Africa.

**Objective:**

This study aimed to assess the proportion of adenomas in northern Nigeria that over-express COX-2.

**Methods:**

This 5-year retrospective, descriptive, hospital-based study examined the COX-2 immunohistochemistry of all histologically diagnosed colorectal adenomas in Aminu Kano Teaching Hospital, Kano, Nigeria, between 01 January 2015 and 31 December 2019. Age, sex, site, diagnosis, and grade were obtained from the Kano cancer registry and slide reviews of cases.

**Results:**

There were cases of 29 adenoma (male, *n* = 20; female, *n* = 9). Adenoma occurred more commonly among male patients (M:F, 2.2:1), in the age group 40–79 years, and included tubular adenomas (62.1%), tubulovillous adenomas (27.6%), and villous adenomas (10.3%). Over-expression of COX-2 was observed in 3.4%. There was no association between COX-2 expression and age, sex, site, histological subtype, or grade.

**Conclusion:**

Over-expressed COX-2 was observed in only 3.4% of adenomas, which may indicate its early involvement in the spectrum of adenoma-carcinoma sequence.

**What this study adds:**

It provides key information about COX-2 expression in adenoma in an African population, which may serve as a rationale for other studies regarding COX-2 targets for chemoprevention and therapy in adenoma and colorectal cancer.

## Introduction

Colorectal adenomas or adenomatous polyps, which are the major sub-types of colorectal polyps, are benign glandular neoplasms of colorectal epithelium that occur either sporadically or as part of a polyposis syndrome, with some having malignant potential. In Nigeria, the proportion of colorectal polyps that are adenomatous ranges from 28.8% to 47.5%.^1,2,3,4,5^ The distinct parameters that make adenoma relevant clinically include being a precursor lesion, harbouring invasive carcinoma, long latency period of malignant transformation, having similar clinical features with colorectal cancer (CRC), and as part of hereditary CRC syndrome.^6,7,8,9^ Emerging evidence implicates the role of the cyclooxygenase-2 (COX-2) enzyme in adenoma. Carriers of the COX-2 A-1195G AG genotype are at a twofold increased risk of developing colorectal adenomas, and 2.9 times increased risk of developing cancer, suggesting a role of COX-2 as a potential biomarker for cancer risk.^10^ Contrastingly, the carriers of COX-2 A-1195G AA genotype have 1.8 times reduced risk of developing adenomas, and 3.1 times reduced risk of developing CRC.^10^ Chronic use of cyclooxygenase antagonists such as nonsteroidal anti-inflammatory drugs, particularly aspirin, is associated with reduced adenoma incidence, aggressiveness, size, and recurrence.^11,12,13,14^ Inhibitors of cyclooxygenases such as nonsteroidal anti-inflammatory drugs are useful in the chemoprevention and management of adenoma, and they exert their effects by inhibiting cyclooxygenases (cyclooxygenase-1 and COX-2), which are enzymes required for the metabolism of arachidonic acid to prostaglandins.^15,16,17^ The expression of COX-2 is highly induced by tumour promoters, mitogens, and cytokines.^15^ Over-expression of COX-2 is associated with inflammation, initiation of cancer, cancer progression, proliferation of surviving cancer stem cells, activation of vascular endothelial growth factor, angiogenesis, invasion, metastasis, and tumour relapse after therapy.^16,17^

The rate of COX-2 over-expression increases during the course of CRC development from adenoma to CRC in situ to invasive CRC, thus recapitulating events in the adenoma-carcinoma progression.^18^ It may, therefore, imply that COX-2 over-expression may be a marker of the progression of CRCs from adenomas.^18^ However, studies on COX-2 and its antagonists in colorectal adenomas are limited in Africa, as most studies are from Asia and Western countries. This study examined COX-2 expression in adenoma in an African population, and it may serve as a rationale for other studies on COX-2 and its target in colorectal adenoma and carcinoma chemoprevention and therapy.

This study aimed to assess the proportion of adenomas in northern Nigeria that over-express COX-2 and establish any relationship between COX-2 over-expression with clinicopathological features such as age, sex, histological subtype, and tumour grade.

## Methods

### Ethical considerations

Ethical approval for this study was obtained from the Health and Research Ethics committee of Aminu Kano Teaching Hospital, Kano (ethical review reference number: AKTH/MAC/SUB/12A/P-3/V1/2904). Additional consent of individuals whose samples were used in this study was waived, as is the institutional ethical committee’s policy for retrospective studies involving non-human subjects. Patients’ identities were concealed at all times, and records were kept under lock.

### Study design and setting

This retrospective, descriptive, hospital-based study was carried out in the Histopathology Laboratory of the Department of Histopathology, Aminu Kano Teaching Hospital, Kano (north-western Nigeria). Aminu Kano Teaching Hospital is a tertiary hospital with over 700-bed capacity. The histopathology department of Aminu Kano Teaching Hospital receives an average of 5500 histological samples per annum, and renders services including cytology, histology, autopsy, and immunohistochemistry.

### Inclusion and exclusion criteria

Adenoma cases included in this study were all cases that were histologically diagnosed with colorectal adenoma in the study centre from 01 January 2015 to 31 December 2019. Cases with insufficient clinical information, particularly biodata, missing or damaged blocks, and tissue blocks with insufficient tissue were excluded (in this regard, excluded case is zero).

### Data collection

Data, such as age, site of lesion, histologic diagnosis, and grade of the disease was obtained from Kano cancer registry, pathology request forms, patients’ case notes, duplicate copies of histopathological reports, and slide reviews of cases. The patient’s identity was concealed at all times. The cases were classified and graded according to the World Health Organization classification of tumours of the colon and rectum.^19^ The two-tiered system of grading was used based on prognosis: low grade and high grade.^19^

### Immunohistochemistry for cyclooxygenase-2 expression

Immunohistochemical staining was performed using anti-COX-2 rabbit polyclonal antibody (Elabscience, Houston, Texas, United States; catalogue No. E-AB-70031), used at a 1:500 dilution according to standard immunohistochemical staining protocols. The antibody was stored at −20 °C (using −25 °C freezer 110 L, Biobase Scientific, Jinan, Shandong, China) and it was centrifuged (using LC-4K-2 Centrifuge, Biobase Scientific, Jinan, Shandong, China) before opening to ensure complete recovery of contents. A kidney sample (an autopsy sample) with intact renal tubules was used as a positive control, while a negative control was obtained by replacing the primary antibody with distilled water.

#### Tissue preparations (formalin-fixed paraffin-embedded tissue blocks)

Formalin-fixed paraffin-embedded tissues retrieved were cut or sectioned at 3 µm using a BK-2218 microtome (Biobase Scientific, Jinan, Shandong, China). The tissue slides were left to dry in an oven for 2 h at 60 °C after microtomy. Then, the slides were dewaxed in xylene (obtained from Central Drug House Ltd, Daryaganji, New Delhi, India) for 1 min, and then passed through decreasing concentrations of alcohol at 100%, 95% and 70% for 1 min each for rehydration of sections.

#### Antigen retrieval or unmasking of antigen sites

Antigen retrieval was done by placing the slides in 100 mL of citrate buffer (pH 6) (obtained from Central Drug House Ltd, Daryaganji, New Delhi, India), and then heating using the microwave (TP-J1-8 microwave, Chongqing TOP Oil Purifier Co. Ltd, Jiulongpo, Chongqing, China) heating retrieval method at 100 °C for 15 min – 20 min. Slides were then washed in distilled water for another 2 min and then washed twice with phosphate buffer (obtained from Central Drug House Ltd, Daryaganji, New Delhi, India) for 3 min each. They were then rinsed in distilled water for 2 min; 3% of hydrogen peroxide was added and left for 10 min. Slides were then washed in running distilled water for 2 min and incubated with a blocking reagent (serum; Elabscience, Houston, Texas, United States) for 10 min.

#### Blockage of endogenous peroxidase

To block non-specific antigen sites, tissue sections were incubated for 1 h in 1.5% bovine serum albumin (obtained from Central Drug House Ltd, Daryaganji, New Delhi, India) at room temperature.^20^

#### Immunostaining procedure

Each slide was wiped with cotton wool to remove excess blocking solution. Incubation with the primary antibodies was carried out at room temperature for 30 min with 200 µL of anti-COX-2 (Elabscience, Houston, Texas, United States; catalogue No. E-AB-70031). Following the primary antibody incubation step, a secondary antibody from a streptavidin-biotin complex peroxidase kit (LSAB+ kit, Dako, Copenhagen, Denmark) was then incubated by the manufacturer’s instructions. Peroxidase activity was also developed with the substrate 3,3-diaminobenzidine tetrahydrochloride (Dako) by incubating the tissue sections in 3,3-diaminobenzidine tetrahydrochloride for a period of 3 min.^20^ The tissue slides were washed in running water for a period of 3 min, and then counterstained with haematoxylin (obtained from Central Drug House Ltd, Daryaganji, New Delhi, India). The tissue sections were then dehydrated in increasing concentrations of alcohol. They were cleared in xylene and the application of cover slip was done using Distyrene Plasticizer Xylene (obtained from Central Drug House Ltd, Daryaganji, New Delhi, India) and they were allowed to dry.

The slides were viewed under the light microscope (DM 500 binocular Leica microscope, Leica Microsystems, Wetzlar, Hessen, Germany), brown cytoplasmic and membranous staining was interpreted as positive staining, and was scored semi-quantitatively using the immunoreactive score system, a final score that is a product of the intensity and distribution of COX-2 immunoreactivity.^20,21,22^ The intensity of staining was scored as 0 for no staining, 1 for weak staining, 2 for moderate, and 3 for strong staining. The percentage of positive tumour cells was scored: 0 indicating no cell with a positive reaction, 1 indicating 1% – 10% of cells with a positive reaction, 2 indicating 11% – 50% of cells with a positive reaction, 3 indicating 51% – 80% of cells with a positive reaction, and 4 indicating greater than 80% of cells with a positive reaction. The final immunoreactive score obtained by multiplying the distribution and intensity for each tumour was graded as follows: 9–12 strongly positive, 5–8 moderately positive, 1–4 weakly positive, and 0 negative. Cyclooxygenase-2 was considered over-expressed if the immunoreactive score is moderate to strong (that is a score of 5 to 12) (Table 1).

**TABLE 1:** Cyclooxygenase-2 immunoreactive scoring system at Aminu Kano Teaching Hospital, Kano, Nigeria, January 2015 – December 2019.

COX-2 expression†	Intensity (I)		Distribution (D)		IRS (IxD)	
	Category	Score	Category	Score	Category	Score
Negative	Non	0	Non	0	Negative	0
Negative	Weak	1	1% – 10%	1	Weak positive	1–4
Positive	Moderate	2	11% – 50%	2	Moderate positive	5–8
Positive	Strong	3	51% – 80%	3	Strong positive	9–12
-	-	-	> 80%	4	-	-

COX-2, cyclooxygenase-2; IRS, immunoreactive score; I, intensity; D, distribution.

† , COX-2 over-expression is IRS of 5–12.

### Statistical analysis

Statistical analysis was performed using Statistical Package for Social Sciences version 25 (IBM^®^ Corp, Armonk, New York, United States). Chi-square was used with statistical significance set at *p* < 0.05 to test the association between the COX-2 expression and clinicopathological parameters such as age, sex, histological subtype, and grade. Proportions and central tendencies of nominal and continuous variables were obtained by descriptive statistics of frequency, mean and median.

## Results

### Age distribution of colorectal adenomas

Twenty-nine adenoma cases were analysed, and 26 cases were endoscopic biopsies. The age distribution of colorectal adenoma cases ranged from 12 years to 80 years with a mean age of 53.4 (standard deviation = 16.0) years. The largest proportion of cases (23 of 29 cases; 79.3%) clustered within the category of patients aged 40 years to 79 years and peaked in the 6th decade. Eleven cases of adenomas (37.9%) occurred before age 50 years while only six cases (20.7%) occurred at age 70 years or more (Table 2).

**TABLE 2:** Age distribution of adenomas with sex, histological sub-types, grade, and site at Aminu Kano Teaching Hospital, Kano, Nigeria, January 2015 – December 2019.

Variable	Age group (years)								Total	
	10–19	20–29	30–39	40–49	50–59	60–69	70–79	80–89	*n*	%
**Sex**										
Male	1	1	1	4	8	1	4	0	20	69.0
Female	0	0	1	3	1	2	0	2	9	31.0
Total	1	1	2	7	9	3	4	2	29	100.0
**Subtype**										
Tubular	1	0	1	5	5	3	2	1	18	62.1
Tubulovillous	0	0	1	1	4	0	1	1	8	27.6
Villous	0	1	0	1	0	0	1	0	3	10.3
Total	1	1	2	7	9	3	4	2	29	100.0
**Grade**										
High	0	1	2	6	3	1	2	1	15	51.7
Low	1	0	0	1	6	2	2	1	14	48.3
Total	1	1	2	7	9	3	4	2	29	100.0
**Site**										
Colon	0	0	2	3	3	2	2	1	13	44.8
Rectum	1	1	0	4	6	1	2	1	16	55.2
Total	1	1	2	7	9	3	4	2	29	100.0

### Gender distribution of adenomas

There were 20 male cases and nine female cases, giving a male-to-female ratio of 2.2:1. Male cases occurred in individuals aged 12 years to 75 years, with a mean age of 51.7 years. The nine cases that were observed among the female patients occurred from 39 years to 80 years, with a mean age of 57.2 years (Table 2).

### Histological types of adenomas

**FIGURE 1:**
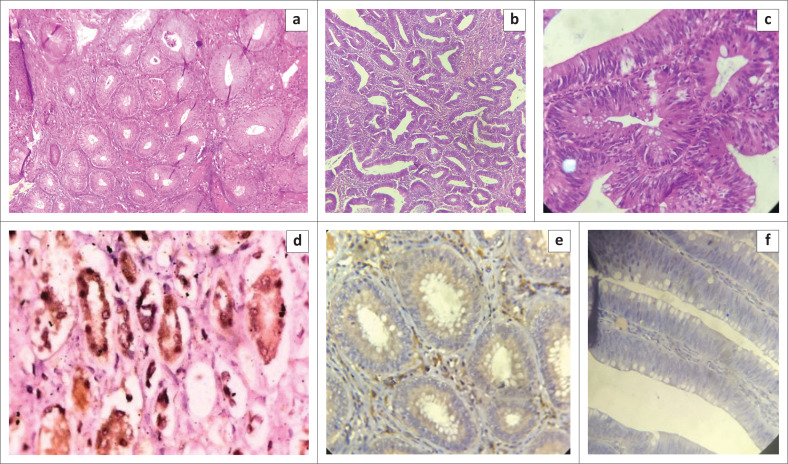
Haematoxylin and eosin and cyclooxygenase-2 Immunohistochemistry photomicrographs of adenoma cases at Aminu Kano Teaching Hospital, Kano, Nigeria. (a) Low-grade tubular adenoma, haematoxylin and eosin ×40, (b) High-grade tubular adenoma, haematoxylin and eosin ×40, (c) low-grade tubulovillous adenoma, haematoxylin and eosin ×100, (d) Cyclooxygenase-2 positive control (renal tubules) ×400, (e) Cyclooxygenase-2 positive (low grade tubular adenoma) ×100, and (f) Cyclooxygenase-2 negative, (low grade tubular adenoma) ×100.

Eighteen (62.1%) cases of colorectal adenoma were tubular adenomas; of these, eight cases were high-grade lesions. Eight (27.6%) cases were tubulovillous adenomas (five of which were high-grade), and only three (10.3%) cases were villous adenomas (including one high-grade lesion, Figure 1). There were no significant associations between histological type and the age group (χ^2^ = 15.8538 degrees of freedom [*df*] = 14, *p* = 0.3224) or sex (χ^2^ = 0.2529, *df* = 2, *p* = 0.8812).

### Grading of adenomas

Fifteen cases (51.7%) of adenoma were high-grade adenomas (Table 2). This comprised nine cases of tubular, five cases of tubulovillous, and one case of villous adenoma. Fourteen cases (48.3%) were low-grade. Among the female patients, there were only five cases (33.3%) of high-grade adenoma, and four cases (28.6%) in the low-grade tumours category. There were no statistically significant associations between the grade of tumour and age (χ^2^ = 8.8808, *df* = 7, *p* = 0.2613) or sex (χ^2^ = 0.0762, *df* = 1, *p* = 0.7818).

### Site of adenomas

Thirteen cases (44.8%) of adenoma were colonic in origin, while the remaining cases were rectal tumours (Table 2). There were no statistically significant associations between the site of tumour and age (χ^2^ = 5.2217, *df* = 7, *p* = 0.6329).

### Cyclooxygenase-2 expression in adenomas

The only case that showed COX-2 positivity (strong positive staining) was a low-grade tubular adenoma in a 60-year-old man (Figure 1). There was no statistical association between COX-2 over-expression and age, sex, or histological type of the tumour.

## Discussion

Adenomas occurred more commonly among male patients (M:F, 2.2:1), in the age group 40–79 years, and they were composed of tubular adenomas (62.1%), tubulovillous adenomas (27.6%), and villous adenomas (10.3%). Over-expression of COX-2 was observed in 3.4%. There was no association between COX-2 expression and age, sex, histological subtype, or grade.

Colorectal adenomas are known precursor lesions for CRC, usually through adenoma-carcinoma sequence, and they also share similar risk factors with CRC.^6,7,8,9^ They, therefore, provide a window for prevention of CRC through screening and early detection of these premalignant lesions.^6^ Early detection of colorectal adenomas through a surveillance screening programme is therefore essential in interrupting the possibility of progression to colorectal cancer.

The adenoma occurrence is more commonly observed in a relatively young population in our society, similar to the age pattern of CRC as demonstrated by a mean age of 53.4 (standard deviation = 16.00) and peak age in the 5th decade in this study. Ibrahim and Dahiru have demonstrated a mean age of 47.3 years among adenomatous polyps in Kano, Nigeria.^3^ A mean age of 47 years has also been observed in China, although the peak age at which adenomas was observed was 65 years.^22^

Adenomas are more frequent in the Western world than in Africa and Asia, but they occur more in older populations in the United States and the United Kingdom, and a mean age as high as 64 years and 68 years has been reported, thereby mirroring the age pattern of CRC in these regions.^23,24^ The possible reason for higher occurrence in the areas may be largely because of exposure to modifiable risk factors such as the Western-type diet similar to that of the CRC. The male preponderance of colorectal adenomas in this study mirrors that of the CRC and has also been reported worldwide.^8,9,25^ It is difficult to explain the higher male affectation but possible reasons have been attributed to differences in access to CRC screening, differences in risk factors exposure, hormonal factors, and socioeconomic factors.^8,9^ It is more likely for men to deposit visceral fat than women, which is a risk factor for both adenoma and CRC.^26,27^ Men are also more likely to have a high consumption of red meat, processed meat, heavy alcohol, and cigarette smoking.^27,28,29^ For histological sub-types and grade, the predominant histological sub-types were tubular adenoma (62.1%), and for the grade, high-grade colorectal adenoma accounted for 51.7% of all grades. Similar findings have also been documented.^3,25^

### Cyclooxygenase-2 expression in adenomas

In the present study, COX-2 expression in the adenomas was 3.4% (1 out of 29 cases). The available data demonstrate relatively lower COX-2 expression in adenoma than in the CRC. The possible reason why COX-2 expression is higher in CRCs than in adenomas may be that although COX-2 expression is involved in tumour initiation, it is also a marker for advanced lesions (from dysplasia in adenomas to frank malignancy and invasiveness in carcinomas).^30,31^ For example, it has been shown that COX-2 expression has a positive correlation with adenoma size, dysplasia, and grade but not with the tumour location or configuration, and the expression is higher in the CRC.^30,32,33,34,35^ This may also explain why the expression of COX-2 in colorectal adenomas demonstrates a wide variation similar to that of the CRC, from 38% to 100%, because of adenoma size, presence of dysplasia, histological subtype, and key genetic mutation.^35,36^ Early adenomas are usually small and the progression of most small adenomas is slow, over several years, even though not all adenomas progress. Some may remain stable, regress or new ones may form. The progression in the spectrum of the adenoma-carcinoma sequence may involve more genetic mutations and molecular interplay, including over-expression of COX-2. To illustrate this, immunohistochemical studies have shown that 37.9% of 39 cases of adenoma were COX-2 positive; 90.9% of these positive cases had a Kirsten rat sarcoma mutation that correlates with tumour size (larger tumour), and the positivity is more frequent in tubulovillous (63.3%) than tubular adenoma (36.4%).^36^ Contrary to this assertion that the COX-2 expression is a marker of progression, a COX-2 immunohistochemical study of nine advanced CRC and 12 adenoma cases in Japan by Bamba et al. showed a higher expression in adenomas than in CRCs.^37^ Bamba et al.’s contrary observation is difficult to decode, but it may stem from the low sample size in their study.^37^

The COX-2 expression (3.4%, 1 out of 29 cases) in this study confirms the assertion that COX-2 is a marker of progression in the adenoma-carcinoma spectrum, but it is relatively lower than other studies. It is difficult to determine the reasons, but the possibilities may include racial factors, as most studies involve non-African subjects, genetic make-up – particularly COX-2 gene polymorphism, and the fact that most adenomas analysed in this current study were colonoscopic biopsies, which may not represent the entire tumour heterogeneity, even though the adenomas from three colectomy cases did not express COX-2 as well. More studies are needed for Africa to explain the possible racial disparity in COX-2 expression in adenomas, because most available COX-2 studies on colorectal adenomas and CRCs are from America and Asian countries. Carriers of the COX-2 A-1195G AG polymorphism have increased risk for CRC and adenoma, and mutation in the adenomatous polyposis coli (APC) gene has been shown to correlate with COX-2 expression in both adenomas and CRCs.^10,38,39^ More studies are needed to establish the role of race in COX-2 expression in adenoma.

### Implications and recommendations

Colorectal adenomas are known precursor lesions of CRC with similar risk factors.^6,7,8^ Thus efforts are required to interrupt the possibility of the progression of adenomas to colorectal cancer.^6^ The expression of COX-2 provides vital information for the uses of nonsteroidal anti-inflammatory drugs in the prevention and treatment of adenoma and hurting adenoma progression into carcinoma. Thus, in terms of the prognosis and COX-2 targeted therapy in the near future, this study provides a strong rationale for additional studies in Nigeria and Africa at large.

### Limitations

The limitation of this study is the inability to include rare histological sub-types of adenoma and relatively low cases of adenomas in this study. To improve this, a multicentre study across Africa will give a better reflection of COX-2 expression in colorectal adenoma in Africa.

### Conclusion

Adenomas occurred twice as commonly in men as in women. Tubular adenomas constituted 62.1% of all adenomas, and 51.7% of adenomas are high-grade adenomas. The rate of over-expression of COX-2 in adenomas is 3.4%, and this may imply its early involvement in colorectal tumourigenesis.
